# Novel Ultra-Sensitive Detectors in the 10–50 μm Wavelength Range

**DOI:** 10.3390/s100908411

**Published:** 2010-09-08

**Authors:** Takeji Ueda, Susumu Komiyama

**Affiliations:** 1 CREST, Japan Science and Technology Agency/5 Sambancho, Chiyoda-ku, Yokyo, 102-0075, Japan; 2 Department of Basic Science, the University of Tokyo/3-8-1 Komaba, Meguro-ku, Tokyo, 153-8902, Japan; E-Mail: skomiyama@thz.c.u-tokyo.ac.jp

**Keywords:** terahertz detector, far-infrared detector, ultra-sensitive detector, photon-counter, single-photon detector, charge-sensitive infrared phototransistor (CSIP)

## Abstract

We have developed novel single-photon detectors in the 10–50 μm wavelength region. The detectors are charge-sensitive infrared phototransistors (CSIPs) fabricated in GaAs/AlGaAs double quantum well (QW) structures, in which a photo-generated hole (+e) in the floating gate (upper QW) modulates the conductance of a capacitively-coupled channel located underneath (lower QW). The excellent noise equivalent power (*NEP* = 8.3 × 10^−19^ W/Hz^1/2^) and specific detectivity (D^*^ = 8 × 10^14^ cm Hz^1/2^/W) are demonstrated for 15 micron detection up to 23 K, which are by a few orders of magnitude better than those of other state-of-the-art high-sensitivity detectors. The dynamic range exceeds 10^6^ (∼aW to pW) by repeatedly resetting the accumulated holes in the upper QW. Simple device structure makes the detectors feasible for array fabrication: Furthermore, monolithic integration with reading circuits will be possible.

## 1. Introduction

Terahertz (THz, ranging 0.3 THz–3 THz in frequency, or 100 μm–1 mm in wavelength) and far infrared (FIR, ranging 20 THz–430 THz, or 15 μm–300 μm) have become intensive, interdisciplinary research fields in recent years [1–3], through long-term fundamental research and application development ground work. Historically, the THz-IR radiation has been researched and applied for heating technology and spectroscopy, but recent research and development are motivated by biology [4], astronomy [5], medicine [6], tele-communication [7], and security [8].

From the viewpoint of spectroscopy, the most remarkable feature of the FIR-THz range is the rich spectra of matter. This makes possible identification of solids, molecules and chemical bonds through *fingerprint*-like spectra. Another important feature is that the FIR-THz radiation is thermally emitted by every material at room temperature [1]. This makes FIR-THz radiation a unique tool for studying the temperature distribution and internal activity of materials. Because of this feature, highly-sensitive detectors have been in demand as the key technology in astrophysics [5]. Recent new technologies also require ultrasensitive detectors: high-resolution passive microscopy [9–11], as well as on-chip devices [12], in which single THz photons are generated, propagated and counted.

Differently from routinely used photon-counters in near-infrared or visible range, photon energies are far smaller (*hν* < 124 meV for *λ* > 10 μm) and the single-photon detection is no longer trivial. In the last decade, a variety of novel detection schemes has been proposed [13–19]. Among them, only semiconductor quantum devices have demonstrated to produce clear single-photon signal against incident FIR-THz photons [14–17]. Charge-sensitive detector (CSIP) [17] is one of those semiconductor devices with single-photon sensitivity, but is far more useful than other ultrasensitive devices from various viewpoints: easy to use, high-speed, wide dynamic range, operable without dilution refrigerator, suitable to make large-array by simple planer structure. In this article the fundamental mechanism of CSIP as well as important experimental results are reviewed.

## 2. Device Structure and Detection Scheme

**Figure 1.**
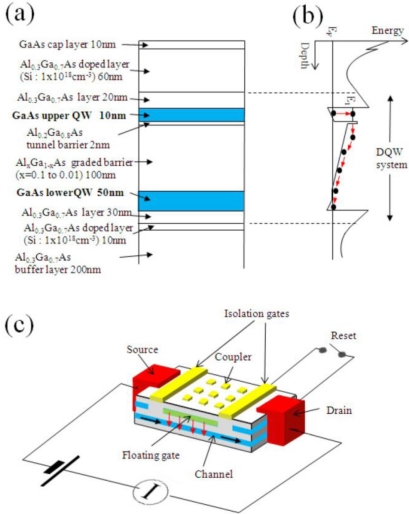
**(a)** Crystal structure for CSIP (*λ* = 15 μm). **(b)** The conduction-band energy diagram of DQW system. **(c)** Schematic representation of a CSIP as a photo-active FET.

The detectors utilize a double-quantum well (DQW) structure (Figure 1(a)), where photo-excited electrons are generated via intersubband transition in the upper quantum well (QW). A photo-excited electron escapes out of the upper QW through the tunnel-barrier, and relaxes into the lower QW as shown in Figure 1(b). Since the upper QW is electrically isolated from the lower QW by negatively biasing the surface metal gates, the isolated upper QW is positively charged up due to the photo-excitation. The pile-up positive charge (hereafter referred to as a “photohole”) in the isolated upper QW is sensitively detected by an increase in conductance through the lower QW as shown in Figure 1(c). In short, the device works as a photo-sensitive field effective transistor with a photo-active floating gate served by the upper QW.

The epitaxial layers are grown by molecular-beam epitaxy on semi-insulating GaAs substrate [17,20–26]: They consist of a 1 μm thick buffer layer (Al_0.3_Ga_0.7_As 20 nm/GaAs 2 nm superlattices), a Si doped (1 × 10^18^ cm^−3^) 10 nm Al_0.3_Ga_0.7_As electron-supply layer, a 30 nm Al_0.3_Ga_0.7_As spacer layer, a 50 nm GaAs lower QW layer, a 100 nm composition graded Al_x_Ga_1−x_As (x = 0.01 → 0.1) barrier layer, a 2 nm Al_0.2_Ga_0.8_As tunnel barrier, a 10 nm GaAs upper QW layer, a 20 nm Al_0.3_Ga_0.7_As spacer layer, a Si doped (1 × 10^18^ cm^−3^) 60 nm Al_0.3_Ga_0.7_As layer, and a 10 nm GaAs cap layer. Typical electron density, *N_s_*, and mobility, *μ*, are around *N_s_* = 3 × 10^11^ cm^−2^ and *μ* = 3 × 10^4^ cm^2^/Vs (for undoped QWs) or 100 cm^2^/Vs (for doped QWs), respectively. The above mentioned structure is for detecting *λ* = 15 μm. For different wavelengths, the width of the upper QW and height of the wall (confining barrier) as well as Si doping density inside QW are tuned so that intersubband transition energy correspond to the target wavelength [27,28].

As illustrated in Figure 1(c) the device consists of a wet-etched DQW mesa, alloyed AuGeNi ohmic contacts, Au/Ti Schottky gates, and Au/Ti photo-coupler. The device is fabricated with standard electron-beam lithography technique. Both of the QWs are connected by ohmic contacts, and can be electrically isolated by biasing metal isolation gates. The photo-coupler is used to cause intersubband transition by generating electric filed normal to the plane of the QW against the normally incident radiation.

**Figure 2.**
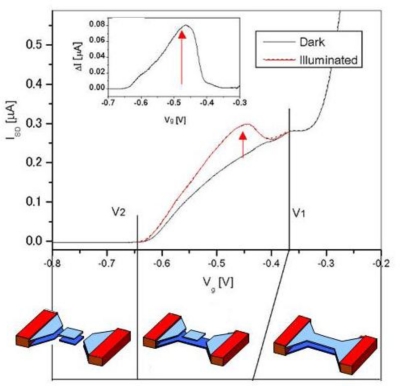
I-V measurement of a CSIP with scanning gate bias Vg. Photo signal appears under illumination when floating gate is formed.

Curves in Figure 2 are the gate-bias-dependent source-drain currents with and without radiation. In dark condition the current decreases with decreasing gate bias V_g_, changing the slope of its decrease at *V_g_* = −0.37 V and completely vanishing at *V_g_* = −0.64 V. The two different slopes correspond to disconnection of each electron layer in the DQW system as schematically shown in bottom of Figure 2. Under illumination the curve increases when upper the QW is electrically isolated (−0.64 < Vg < −0.37).

This photoresponse can be interpreted by increase of electron density in lower QW induced by capacitively coupled photoholes stored in the isolated upper QW. The unit increment of current *I_e_* induced by one photohole in the isolated upper QW (area of *L* × *W*) is given by [17,22]:

(1) $$I_{e}=\frac{e\mu V_{\mathrm{SD}}}{L^{2}}$$

where *e* is the unit charge, *μ* the electron mobility of lower QW, *V_SD_* the souce-drain (SD) voltage, *L* the length of constricted channel. For example, unit increment *I_e_* = 3 pA is given for *μ* = 1 m^2^/Vs, *V_SD_* = 10 mV and *L* = 16 μm. The signal *I_e_* persists as long as a photohole stays in upper QW. By setting the lifetime *τ* = 1 s, the amplification factor, or photoconductive gain, is given as *G* = *τI_e_*/*e* = 1.8 × 10^7^. This value is comparable to that of photomultiplier tubes. The increase of the current is proportional to the number of accumulated photoholes *p*:

(2) $$\Delta I={\mathrm{pI}}_{e}$$

**Figure 3.**
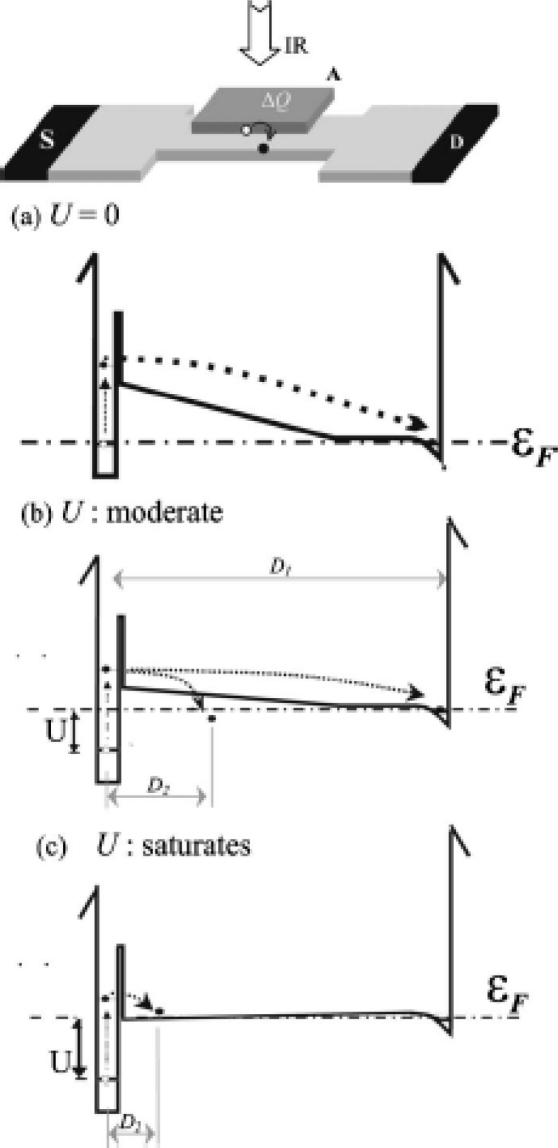
Energy diagram illustrating saturation of photosignal; **(a)** No deformation in initial/dark condition. **(b)** Moderate deformation in the linear response regime. **(c)** Strong deformation in the saturated regime.

Under steady illumination, *p* is a linearly increasing function of time if the lifetime of photoholes is longer than the relevant time of integration. The number of photoholes, of course, does not increase infinitely, but reach a saturated value as in Figure 3. The saturation is caused by balance between generation and recombination speeds of photoholes which change with deformation of the potential profile due to accumulating positive charges in upper QW as illustrated in Figure 3 [21]. The potential drop is given by *U* = *pe*^2^*d*/*ɛLW*, where *d* = 150 nm is the distance between upper and lower QWs, *ɛ* = 12 × (8.85 × 10^−12^) F/m is the electric permittivity of GaAs.

The number of photoholes is described by the rate equation:

(3) $$\frac{\mathrm{dp}}{\mathrm{dt}}=\eta \Phi -\frac{p}{\tau }$$

where *η* is quantum efficiency, Φ is the incident photon flux, and *τ* is the lifetime of photoholes. The first and second term in the right-hand side of Equation 3 refer to the generation and recombination speed of photoholes, respectively.

## 3. Device Operation and Photo-Signal

**Figure 4.**
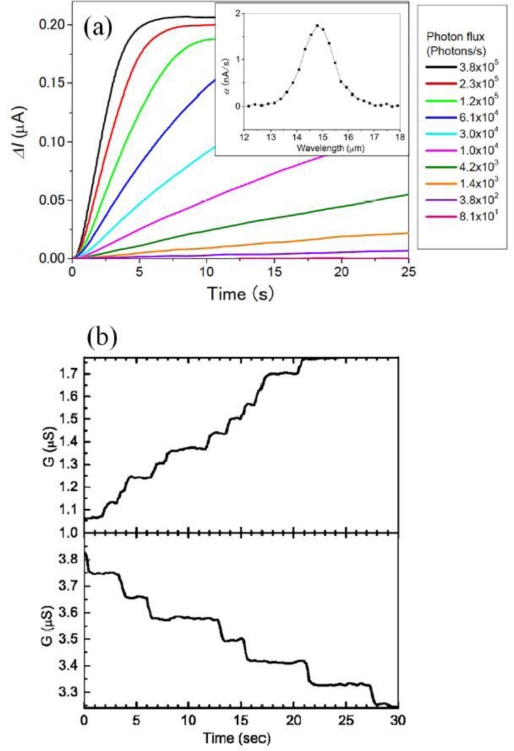
**(a)** Time-traces of the photo current obtained in different incident radiation intensities. The inset displays the spectrogram. **(b)** (Upper panel) Stepwise conductance increase seen under a weak radiation. (Lower panel) Stepwise conductance decrease seen in the dark after the measurement of the upper panel.

Figure 4 displays the photo current time traces taken at 4.2 K with a fixed surface gate bias *V_g_* = −0.45 V applied at *t* > 0 s. The gate bias voltage is determined by the experiment in Figure 2. The curves in Figure 4(a) are taken with different radiation intensities. The signal curves show linearly increasing region and following saturated region. This behavior is clearly explained by Equation 3 and Figure 3: In the initial and moderate state in Figures 3(a,b), the lifetime *τ* is large enough to neglect second, recombination term (*p*/*τ* ≈ 0) and, therefore, system follows *dp*/*dt* = *ηΦ*. With increasing number of *p*, recombination term increase, and finally the system reach saturation (*dp*/*dt* = 0).

The signal for extremely weak radiation, which is the case of lowest photon flux in Figure 4(a), is given as stepwise increase as shown in the upper panel of Figure 4(b) [22]. The step height of *ΔG* ≈ 0.05 μS is close to the value of *I_e_*/*V_SD_* = 0.07 μS estimated from Equation 1 for active area *A* = *LW* ≈ 1.5 × 0.8 μm^2^ and the mobility of lower channel *μ* ≈ 1 × 10^4^ cm^2^/Vs. This good agreement assures that each step corresponds to the single photon signal. Under dark condition, the signal then showed stepwise, slow decrease by hole-electron recombination (lower panel in Figure 4(b)). The results demonstrate extremely long lifetime of photo-generated holes (up to hours).

In the linear region, the photo signals of CSIPs appear as integrated single photon signals, where the slope is proportional to the photon flux via Equations 1–3: *dI*/*dt* = *I_e_ηΦ*. However, the devices immediately reach saturation, and are no more sensitive. In practical application of CSIPs, therefore, it is very important to expand the dynamic range.

**Figure 5.**
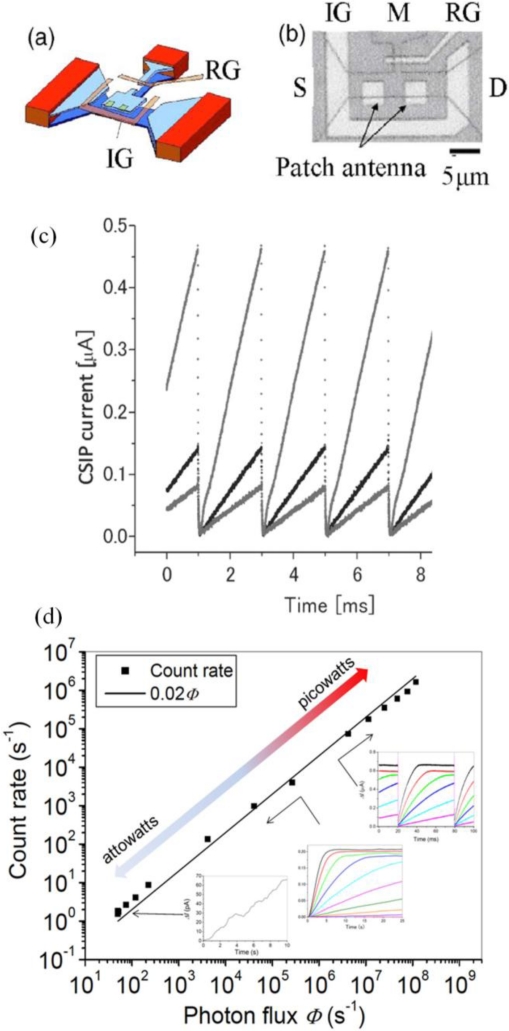
**(a)** Schematic representation of a CSIP with reset gate. IG is the isolation gate and RG is the reset gate. **(b)** A microscopic image of the device with 16 × 4 μm^2^ isolated island formed by the upper QW. **(c)** Reset signal for different radiation intensities. **(d)** Count rate of the photo signal *vs.* incident photon flux *Φ*.

An effective method for extending the dynamic range is introducing the reset function to CSIP [23]. When a short positive pulse (1 μs-duration) is applied to an additional resetting gate (RG) in Figures 5(a,b), the accumulated charge on the isolated QW is released to the ground, and thereby resetting the device in the highly photosensitive state. The potential profile oscillates between (a) and (b) in Figure 3 by the reset operation and resulting in the linear response shown in Figure 5(c). In Figure 5(d) we plot count rates, given by dividing linear slope *dI*/*dt* by unit signal amplitude *I_e_*, *versus* photon flux *Φ* = 5 × 10^1^–1 × 10^8^ tuned by the temperature of blackbody radiation source described in the next section. The original time traces are also shown in the inset of Figure 5(d). The dynamic range is extended to exceed 10^6^, where the upper limit is not given by detector but by the emitter used in the experiment [17].

Recently CSIPs for several different wavelengths are demonstrated [27,28] in 10–50 μm range (realized in 12, 15, 27, 29, 45 μm). The wavelengths are tuned by confinement of upper QW *i.e.*, width of well and height of barriers of QW. The mechanism and operation are exactly same as *λ* = 15 μm described above.

## 4. Figures of Merit

**Figure 6.**
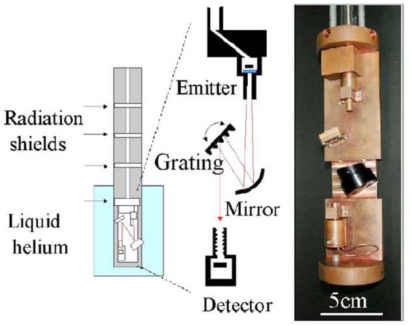
All-cryogenic spectrometer, schematic representation and a photograph.

The quantum efficiency *η*, responsivity *R* and specific detectivity D^*^ of CSIP (*λ* = 15 μm) are determined accurately in all-cryogenic spectrometer [17] shown in Figure 6. In order to perform experiments without background blackbody radiation (BBR), the spectrometer is shielded by a stainless steel pipe and kept in liquid helium. The system includes glass-covered 1 kΩ thin metal chip resistor suspended in a vacuum chamber, serving as BBR source by Joule heating, and a diffraction grating to produce monochromatic photon flux. The system is necessary to accurately determine figures of merit of ultrasensitive detectors in FIR-THz range because thermal radiation from 300 K optical elements is catastrophically strong. Spectrogram and time-traces in Figure 4 (a) and Figure 5 (d) are taken by this setup. Given photon flux is accurately determined by Planck’s formula with the temperature of BBR emitter monitored by a thermocouple.

The external quantum efficiency *η*, which includes both the coupling efficiency and absorption efficiency, directly determined to be 2% from Figure 5(d). Responsivity is the photo current amplitude divided by the incident power: *R* = *ΔI*/*P* = *ηI_e_τ_r_/hν* = 4 × 10^4^–4 × 10^6^ A/W by noting *η* = 0.02, *I_e_* = 3 pA, *hν* = 83 meV, and the reset interval *τ_r_* = 1 × 10^−3^ to 1 s.

Noise-equivalent power in photon-counting device is given as *NEP* = *hν* (2*Γ*)^1/2^/*η*, where *Γ* is the dark count rate. The experimentally determined value is *NEP* = 6.8 × 10^−19^ W/ Hz^1/2^ from *Γ* = 0.5.

**Figure 7.**
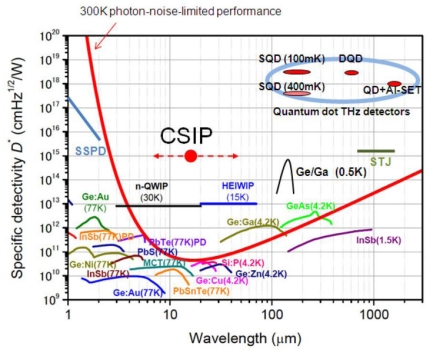
Spectral response characteristics of various infrared detectors.

Specific detectivity is the reciprocal value of NEP normalized by active area *A* of the detector: D^*^= A^1/2^/*NEP*. With *A* = 64 × 10^−8^ cm^2^, we have D^*^ = 1.2 × 10^15^ cm H^1/2^/W. This value is compared with other conventional detectors in Figure 7. Those values are by a few orders of magnitude better than those of the state-of-the-art high-sensitivity detectors. This values even improved by improving described *η* in the next section.

## 5. Photo-Couplers for Higher Quantum Efficiency

Intersubband transition arises by electric field perpendicular to the QW plane by the selection rule. We need, therefore, photo-coupler converting direction of electric field of normal incident radiation which is parallel to the QW plane. Among excellent figures of merit of CSIPs, the quantum efficiency is limited to 2%. The relatively low efficiency arises from a low optical absorption in a single QW layer, which is in contrast with ∼50% of multi-QW infrared photodetectors (QWIPs) [29].

**Figure 8.**
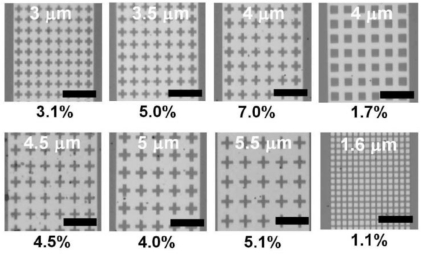
Photo couplers. The period is marked in each micrograph. The scale bar indicates 10 μm. Numbers below each structure are the experimentally derived values of the quantum efficiency *η* [25].

High efficiency of QWIPs is realized by the stacked QW layers (more than 30 QWs at a depth of ∼1 μm) as well as optimized photo-couplers, e.g., gratings coupled with waveguides. The photo-coupler geometries used in QWIPs, however, cannot be directly applied to CSIPs because a CSIP has only one QW for detection, which lies at a depth of 100 nm beneath the surface. Recently we proposed and demonstrated efficient photo-couplers for CSIPs (*λ =* 15 μm) by exploiting surface-plasmon-polariton (SPP) resonance occurring in aperture metal sheets coated on top of the crystal surface (Figure 8) [25]. The SPP resonance induces wavelength-selective strong electric field confined near the surface of the metal sheets, which effectively intensifies the subband transition in the QW 100 nm below the surface. Cross-shaped hole arrays yield the highest efficiency of *η* = 7%, which is by a factor four higher than that of the square-metal-pad arrays. The improved quantum efficiency directly improves figures of merit descried in the previous section.

## 6. Temperature Dependence

Higher temperature operation is desired for practical applications. There is in general, however, a trade-off between high sensitivity and operation temperature. At the elevated temperature devices start to emit BBR or lose their sensitivity by too many thermal excited electrons overwhelming photo-excited ones.

**Figure 9.**
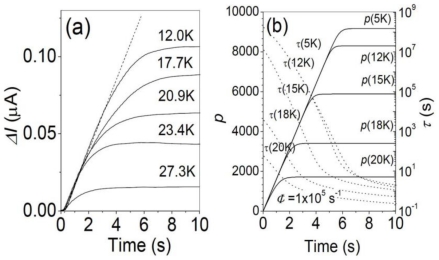
**(a)** Time traces of the photocurrent with *Φ* = 1 × 10^5^ s^−1^ at different temperatures. **(b)** Theoretical time traces of photo current in terms of *p* at different *T* (solid line), and lifetime change (dotted line) [24].

In Figure 9(a), time traces of photo-current at different temperatures *T* are displayed under the fixed photon flux *Φ* = 1 × 10^5^ s^−1^ [24].

The temperature effect appears as the lower amplitude of photo-current saturation. It should be noted that the slope, *dI*/*dt* = *η*Φ*I_e_*, in the initial stage of each trace is independent of *T*, assuring that *ηI_e_* is independent of *T*. This means higher frequency reset operation is required, *i.e.*, the integration time is shortened, in the elevated temperatures. The photo-signal is discernible up to 30 K for the CSIP of *λ* = 15 μm. The derived *NEP* and D^*^ up to *T* = 23 K with integration time of 1 s are given as *NEP* = 8.3 × 10^−19^ W/Hz^1/2^, and D^*^ = 9.6 × 10^14^ cm Hz^1/2^ /W [24], which are not very different from the 4.2 K values given at 4.2K (Section 4, [17]).

As mentioned in Section 2, the photo-current saturation occurs when the recombination process becomes equivalent to the photohole generation process. The lower level saturation at elevated temperatures is also understood by enhanced recombination: more thermally excited electrons in the lower QW contribute to the recombination process. Finally at around *T* = 30 K, the number of traveling electron across the barrier exceeds that of photo-emitted electrons even with no band-deformation, *i.e.*, no photohole stored in upper QW.

This phenomenon can be successfully explained by a simple model by thermionic electron emission [24] which gives an expression of recombination term *p*/*τ* in Equation 3. As shown in Figure 5(b), the predicted time traces of *p* (photo-signal) at different temperatures substantially reproduce experimental results. The lifetime is also estimated as in Figure 5(b). It should be noted that adjustable parameters are not involved in the calculation. The model also predicts that operable temperature limit (*T* = 30 K for *λ* = 15 μm) is proportional to photon energy to be detected [24].

## 7. Conclusions

We developed novel ultrasensitive detectors in a 10–50 μm wavelength range. To our knowledge, single-photon detection is achieved in this spectral range for the first time. The accurately determined specific detectivity for CSIPs detecting *λ* = 15 μm is a few order magnitudes superior to those of other detectors and it persists up to 23 K. Although CSIP has only one QW layer for photo absorption, the quantum efficiency has improved up to 7% by using metal mesh photo-coupler. CSIPs are featured by not only ultra-high sensitivity, but also by versatile applicability due to higher temperature operation as well as extremely wide dynamic range. The simple planar structure is feasible for array fabrication including future monolithic integration with reading circuit.

##
