# Supplementary figures and images for: Novel Ultra-Sensitive Detectors in the 10–50 μm Wavelength Range
Source: Sensors (Basel). 2010 Sep 8;10(9):8411–23. doi: 10.3390/s100908411 (PMC3231243; doi:10.3390/s100908411)

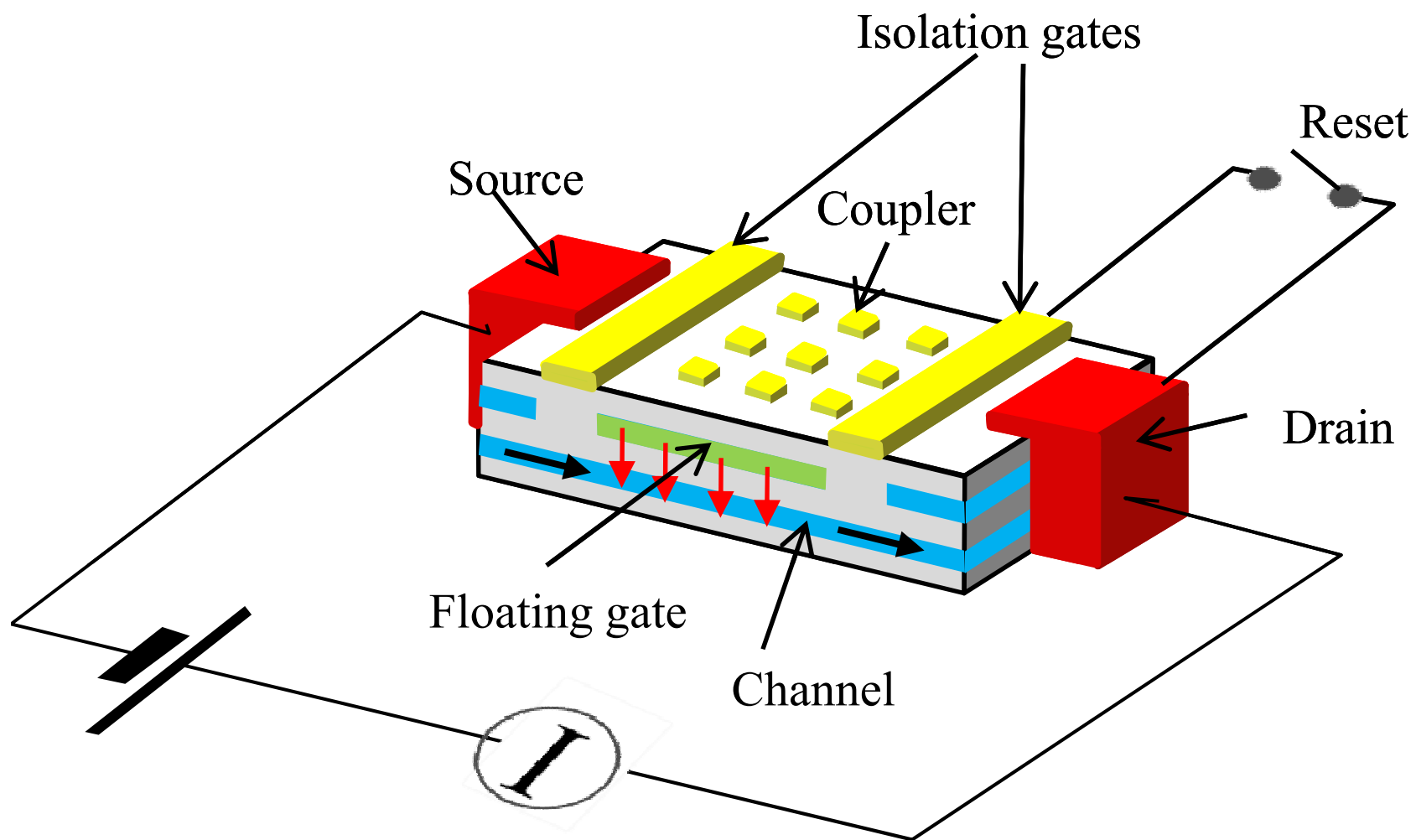

Supplement: Supplementary file 1 [file sensors-10-08411-s001.pdf]
